# The predictive and prognostic role of baseline 2-[18F]FDG PET/CT volumetric and dissemination features in classical Hodgkin lymphoma

**DOI:** 10.1007/s00259-026-07862-x

**Published:** 2026-03-25

**Authors:** Domenico Albano, Alessandro Re, Rosa Daffini, Alessandra Tucci, Francesco Bertagna

**Affiliations:** 1 Nuclear Medicine, University of Brescia, Brescia, Italy; 2 Nuclear Medicine Department, ASST Spedali Civili di Brescia, Brescia, Italy; 3 Hematology Division, ERN/EuroloodNet, ASST Spedali Civili di Brescia, Brescia, Italy; 4 Nuclear Medicine, Spedali Civili di Brescia, P.le Spedali Civili 1, Brescia, 25123 Italy

## Abstract

**Background:**

This study aimed to evaluate whether baseline 2-deoxy-2-[18F]-fluoro-D-glucose (2-[^18^F]FDG) positron emission tomography/computed tomography (PET/CT) features reflecting tumor burden (Metabolic Tumor Volume [MTV] and Total Lesion Glycolysis [TLG]) and disease dissemination (Dmax) can predict treatment response and prognosis in patients with Hodgkin Lymphoma (HL).

**Methods:**

A retrospective single-center study was conducted on 361 classical HL consecutively diagnosed and treated with ABVD from January 2007 to June 2023. All patients underwent baseline, interim (iPET), and end-of-treatment (eotPET) PET/CT scans. Baseline MTV, TLG, and Dmax were calculated using specialized software (LIFEx 7.7). Treatment response was evaluated using Deauville scores and corresponding Lugano criteria. The primary endpoint was to evaluate the role of PET features in predicting progression-free survival (PFS). PFS was analyzed using Kaplan-Meier and Cox proportional hazards models.

**Results:**

Baseline MTV, TLG, and Dmax were significantly higher in patients who failed to achieve a complete metabolic response at both interim and end-of-treatment assessments (*p* < 0.001). Multivariate analysis identified iPET metabolic response, eotPET metabolic response and baseline MTV, TLG, and Dmax as independent prognostic factors for PFS. Combining MTV and Dmax allowed for better risk stratification; Patients with both low MTV(≤ 111 cm^3^) and low Dmax (≤ 38 cm) had a median 5-year PFS of 90%, compared to 39% for those with both high MTV and high Dmax.

**Conclusions:**

Baseline 2-[18 F]FDG PET/CT metabolic parameters expressing tumor burden and dissemination are strong, independent predictors of treatment response and prognosis in HL. Integrating these features provides a robust method for early risk stratification.

**Supplementary Information:**

The online version contains supplementary material available at 10.1007/s00259-026-07862-x.

## Introduction

Classical HL (cHL) represents approximately 10% of all lymphoma and despite an optimal 5-year relative survival with modern therapy, the risk of relapse or progression is not negligible (about 20%) as the risk of long-term treatment toxicity [1]. 2-deoxy-2-[18F]-fluoro-D-glucose (2-[^18^F]FDG) positron emission tomography/computed tomography (PET/CT) is a hybrid imaging tool with strong evidence in ameliorating staging and evaluation of treatment response in FDG-avid lymphoma, including HL, Follicular Lymphoma (FL) and Diffuse Large B cell Lymphoma (DLBCL) [2, 3]. For the evaluation of treatment response, specific criteria were introduced a decade ago, the Lugano criteria, which include a 5 point scale, known as the Deauville score (DS) and were applied both at interim and end-of-treatment PET/CT (iPET/CT and eotPET/CT) [2, 3]. Beyond visual scores, also semiquantitative parameters derived from PET images at baseline were studied with promising findings, especially in the prognostication [4]. These variables encompass various disease characteristics, including tumor burden, represented as metabolic tumor volume (MTV) and total lesion glycolysis (TLG), and features of dissemination, such as Dmax, which measures the distance between areas of increased uptake. Concerning MTV and TLG, several evidence of prognostic significance are available [5], but most of these studies are retrospective, based on small and/or heterogeneous populations and different methodologies applied. Instead, about Dmax, only a few studies on HL have been reported [6–9] with controversial results. The consequence is that despite extensive development efforts, these semiquantitative PET-based biomarkers remain largely excluded from risk-adapted treatment approaches and are still limited only in the research field.

The aim of this research was to investigate whether the metabolic baseline 2-[^18^F]FDG PET/CT features expressing tumor burden and dissemination may help to predict treatment response (at interim and end of treatment) and prognosis in cHL. Furthermore, we aimed to investigate whether combining volumetric (MTV) and dissemination (Dmax) parameters could generate a more powerful, integrated prognostic model than either parameter alone.

## Materials and methods

### Patients

**Fig. 1:**
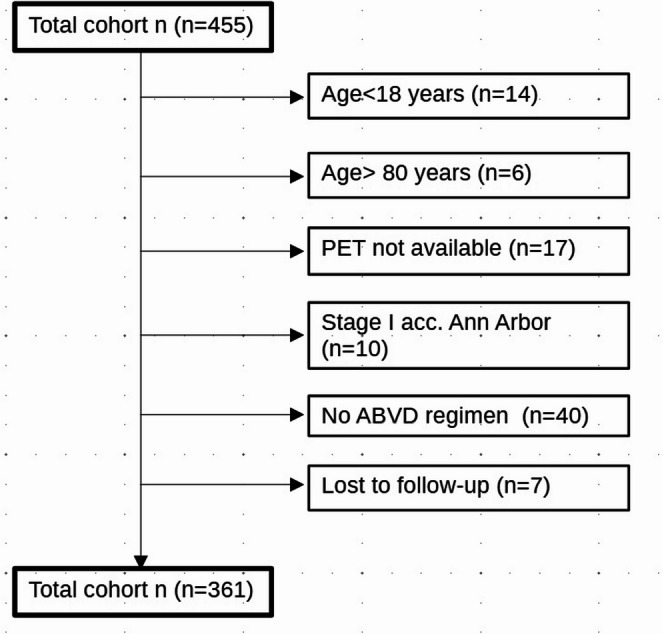
Flow-chart of patients included in the research

This research was a single-center retrospective study and was approved by the ethics committee of CET Lombardia 6, Italy (NP 6211). We included consecutive patients with histologically confirmed cHL diagnosed in our hospital between January 2007 and June 2023, who performed baseline 2-[^18^F]FDG PET/CT (bPET/CT), interim 2-[^18^F]FDG PET/CT (iPET/CT) and end of treatment 2-[^18^F]FDG PET/CT (eotPET/CT) and with a minimum follow-up of 12 months from the diagnosis, homogeneously treated with ABVD/BV-AVD iPET was performed after 2 cycles of chemotherapy, while eotPET was performed after 4 or 6 cycles according to stage and risk factors. Patients with positive iPET/CT switched to a more intense regimen consisting of 4 cycles of BEACOPP regimen and eotPET was considered at the end of the last cycle of BEACOPP. Baseline evaluation for each subject included demographic details (age, gender) alongside key clinical markers such as IPS score, LDH, Ann Arbor stage, and B symptoms + Bulky. We also recorded treatment types and morphological features, specifically identifying bulky disease. Following established criteria, this bulky disease was defined as any mass measuring at least 10 cm or a mass whose diameter was higher than 33% of the internal transverse diameter of the thorax. All patients were treated according to the institution’s standard protocol with a chemotherapy regimen consisting of ABVD (Doxorubicin, Bleomycin, Vinblastine, Dacarbazine). Radiotherapy following chemotherapy was executed when indicated according to the clinical stage, bulky mass or residual disease. Exclusion criteria were; patients younger than 18 years or older than 80 years; patient with stage I according to Ann Arbor disease (due to the impossibility to calculate Dmax); patients receiving first-line chemotherapy regimen different from ABVD and ABVD-like (including BCVPP, COMP, MBVD, BEACOPP), lack of iPET and/or eotPET, and loss of follow-up; patients with early-stage favorable HL who received 2 cycles of ABVD followed by radiotherapy (Fig. 1).

### 2-[^18^F]FDG PET/CT imaging and interpretation

 2-[^18^F]FDG PET/CT scanning was performed according to international guidelines [10]. The i.v. injection of 2-[^18^F]FDG (activity: 3.5–4.5 Mbq/Kg) was performed after a minimum of 6 h fasting period and with blood glucose levels lower than 150 mg/dL. 2-[^18^F]FDG PET/CT was acquired about 60 min after the radiopharmaceutical injection. Acquisition was done from the skull basis to the mid-thigh. The tomographs available during patient’s recruitment were a Discovery ST and a Discovery 690 (General Electric Company—GE^®^—Milwaukee, WI, USA) with standard parameters (CT: 80 mA, 120 Kv without contrast; 2.5–4 min per bed-PET-step of 15 cm; matrix of 128 × 128 or 256 × 256 and a field of view of 60 cm). bPET/CT was performed prior to any treatment and not earlier than 7 days prior to the first cycle of chemotherapy; iPET/CT was executed during the week before the third cycle (range 1–7 days) and eotPET/CT was performed at least 3 weeks after the completion of the last cycle of chemotherapy.

2-[^18^F]FDG PET/CT scans for all patients were visually and semiquantitatively revised by an expert nuclear medicine physician (DA) who was blinded to the patient anamnesis and outcome. For qualitative analysis, every focal FDG uptake different from physiological distribution and background was considered suggestive of disease. iPET and eotPET scans were classified according to DS and interpreted according to Lugano criteria [2, 3]. According to DS, 2-[^18^F]FDG PET/CT was interpreted as follows: 1 = no uptake above background, 2 = uptake equal to or lower than mediastinum, 3 = uptake higher than mediastinum and lower than liver, 4 = uptake moderately increased compared to the liver, and 5 = uptake markedly increased compared to the liver. With respect to the DS, 2-[^18^F]FDG PET/CT scans were considered as complete metabolic response (CMR) for scores 1–3 and as not complete metabolic (nCMR) response for scores 4–5 (including partial response and refractory/progressive disease). For semiquantitative analysis, we derived the MTV, TLG, and Dmax. MTV was calculated in bPET using LIFEx software version 7.7 [11] and defined using the SUVmax threshold of 4. A fixed SUV threshold of 4.0 was applied to all lesions, irrespective of their individual SUVmax, in accordance with recent consensus recommendations for lymphoma [23]. TLG was calculated as MTV × SUVmean. Dmax was defined as the maximum distance between the two farthest PET-positive lesions, measured in three-dimensional space using the LIFEx software.

### Statistical analysis

Statistical analyses were performed using MedCalc version 19 (MedCalc Software Ltd, Ostend, Belgium). Categorical variables were expressed as absolute and relative frequencies. Continuous variables were summarized using mean, standard deviation, median, minimum, and maximum values. The Mann-Whitney U test was employed to compare baseline metabolic parameters (MTV, TLG, and Dmax) between patients not complete and complete metabolic responses, both at the interim assessment and at end of treatment. Receiver operating characteristic (ROC) curves were generated to determine the optimal cutoff values for MTV, TLG, and Dmax in predicting progression free survival (PFS). Survival curves were estimated using the Kaplan–Meier method and compared via the log-rank test. PFS was defined as the time from diagnosis to the date of first recurrence, disease progression, or death from any cause. To identify independent predictors of PFS, univariate analyses were preformed. Statistical significance was defined by a two-sided p value < 0.05, and results were reported with 95% confidence intervals (CIs). Predictors were entered into the multivariable Cox proportional hazards models if they demonstrated statistical significance (*p* < 0.05) in the univariate analysis. Due to the high biological and statistical correlation between MTV and TLG (collinearity), these variables were not entered into the same multivariable model simultaneously. Instead, we performed separate multivariable models for MTV and TLG to independently confirm their prognostic value. The proportional hazards assumption for the Cox models was verified using Schoenfeld residuals to ensure the validity of the hazard ratios over time. To minimize the risk of overfitting, we maintained an adequate ratio of events (108 progression/relapse events) to the number of covariates included in the final multivariable models.

## Results

### Population features and PET/CT findings

Finally, 361 patients with histological confirmed HL were retrospectively recruited (Fig. 1). One hundred and eighty (49.9%) were male and 181 (50.1%) female; average age was 41.9 years with a range of 18–80 years. Patients were staged according to the Ann Arbor system as follows: stage II (*n* = 149), stage III (*n* = 101) and stage IV (*n* = 111). B-symptoms and bulky disease were described in 142 and 98 patients, respectively. LDH level was high in 172 patients and IPS score was superior or equal to 4 in 140 patients.

bPET/CT scans showed the presence of at least one lesion with increased 2-[^18^F]FDG uptake in all patients, confirming FDG-avid disease in all cases. Mean MTV was 151 cm^3^ (1.6–1708 cm^3^), mean TLG was 1258 (8-22628) and mean Dmax was 36.1 cm (3–93) (Table 1).

**Table 1:** Baseline characteristics of our cohort (n=361)

	Patients, *n* (%)	Mean ± SD	Median (range)
Gender male	180 (49.9%)		
Gender female	181 (50.1%)		
Age at diagnosis, years		41.9 ± 18	40 (18-80)
Stage at diagnosis (Ann Arbor)			
II	149 (41%)		
III	101 (28%)		
IV	111 (31%)		
B symptoms	142 (39%)		
Bulky disease	98 (27%)		
IPS score>3	140 (39%)		
LDH elevated	172 (48%)		
MTV, cm^3^		151 ± 206	76 (1.6-1708)
TLG		1258 ± 2096	410 (8-22628)
Dmax, cm		36.1 ± 17.2	31 (3-93)

*SD *standard deviation, *IPI *international prognostic score, *LDH *lactate dehydrogenase, *MTV *total metabolic tumor volume, *TLG *total lesion glycolysis, *Dmax*, maximum tumor dissemination

## Role of 2-[^18^F]FDG PET/CT in predicting treatment response

Based on DS and Lugano criteria, 217 (60%) patients had CMR and 144 (40%) patients had nCMR at iPET/CT and switched from ABVD to BEACOPP treatment. Instead, at eotPET/CT 292 (80%) patients had CMR and the remaining 69 (20%) nCMR. Among these 69 nCMR, partial metabolic response was registered in 38 cases and progression of disease in 31 cases. Concerning iPET/CT, MTV, TLG and Dmax were significantly lower in patients that reached CMR compared to nCMR (*p* < 0.001 in all cases; Table 2). Accordingly, also at eotPET/CT MTV, TLG and Dmax were significantly lower in CMR patients than nCMR category (*p* < 0.001 in all cases; Table 2).

**Table 2:** Comparison of baseline metabolic features between no complete and complete response groups at interim and end-of-treatment PET/CT

Feature (mean)	Interim PET/CT			End-of-treatment PET/CT		
	CMR *n*=217	Not CMR *n*=144	*p* value	CMR *n*=292	Not CMR n=69	*p* value
MTV, cm^3^	108.3	224	<0.001	125.5	245.9	<0.001
TLG	817.4	2000	<0.001	990.2	2413	<0.001
Dmax, cm	33.5	40.3	<0.001	33.9	44.6	<0.001

*CMR *complete metabolic response, *MTV *total metabolic tumor volume, *TLG *total lesion glycolysis, *Dmax *maximum tumor dissemination

### Prognostic role of 2-[^18^F]FDG PET/CT in predicting progression free survival

**Fig. 2:**
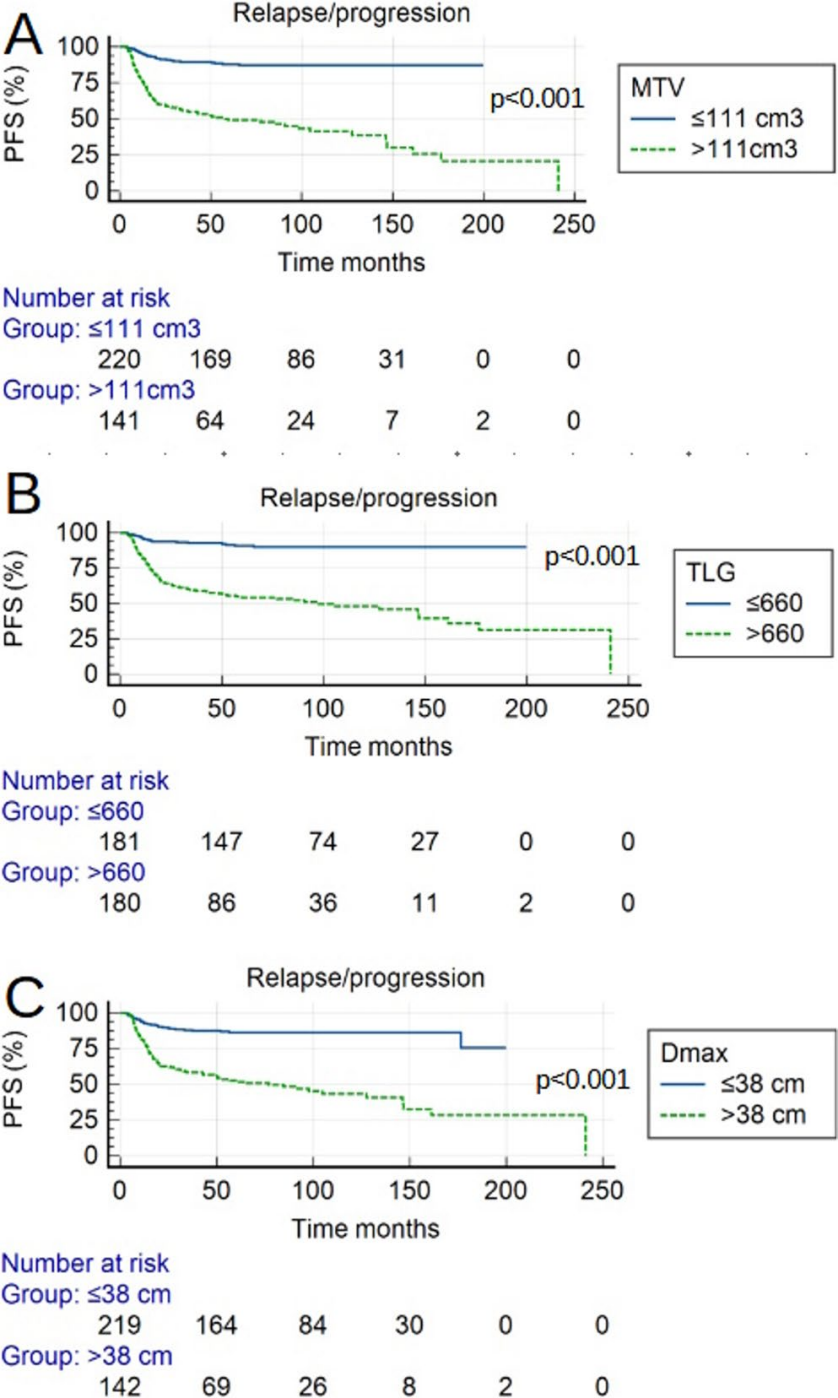
Progression-free survival graphs according to baseline MTV (**A**), TLG (**B**) and Dmax (**C**)

**Fig. 3:**
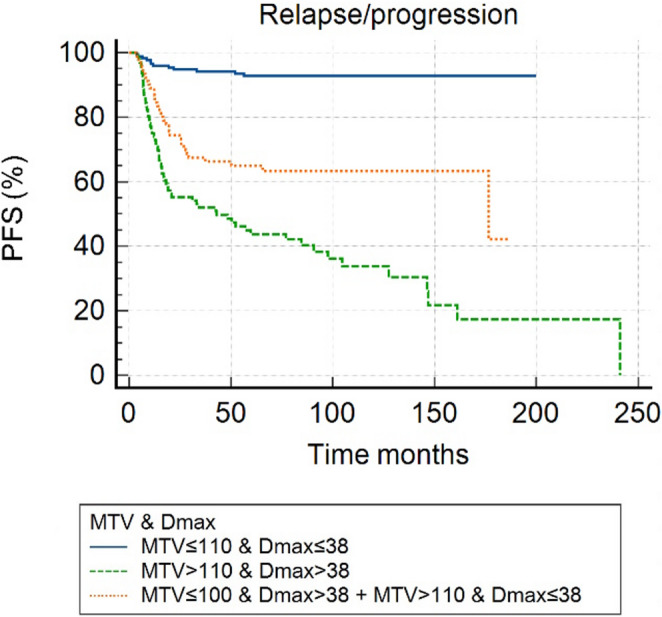
Progression-free survival curves according to the combination of baseline MTV and Dmax

**Fig. 4:**
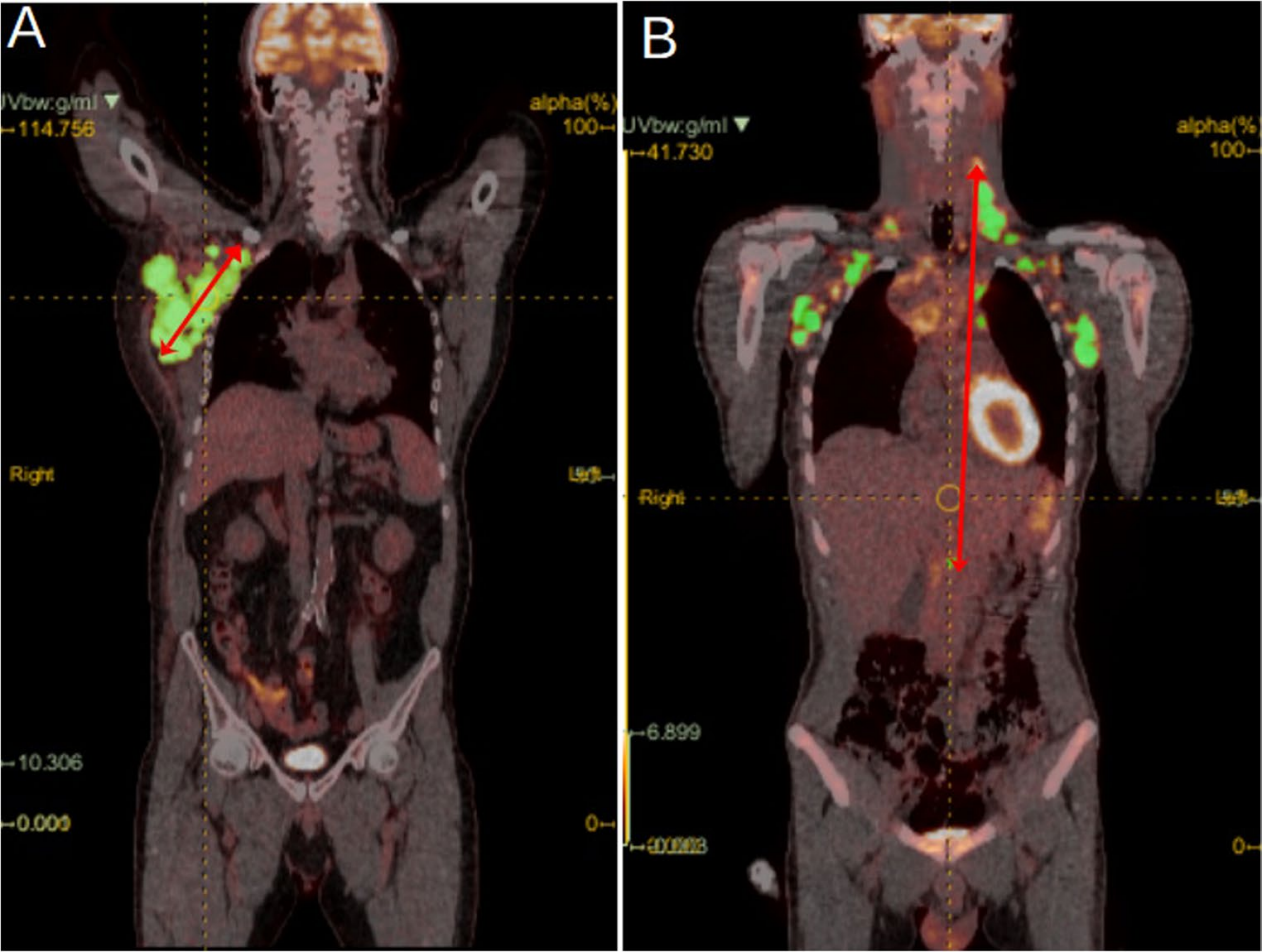
Two representative cases illustrate patients with a similar metabolic tumor volume (MTV) but markedly different disease dissemination. Patient A: A 56-year-old man with stage III disease and a mass consisting of several lymph nodes with increased FDG uptake in the right axilla. Total MTV was 490 cm³ and Dmax was 16.3 cm. Patient B: A 35-year-old male with stage IV disease and multiple areas of increased uptake corresponding to nodes above and below the diaphragm. Total MTV was 452.6 cm³ and Dmax was 67 cm. While the first patient experienced no relapse during the follow-up period, the second patient suffered a recurrence 12 months after the initial diagnosis

At a median follow-up of 79 months (range 12–180), relapse or progression of disease occurred in 108 (30%) cases with an average time of 30 months (range: 4–146 months) from the bPET/CT. The median PFS was not reached for the entire cohort, as more than 50% of patients remained event-free during the observation period. For the prognostic evaluation, MTV, TLG and Dmax were dichotomized using ROC test and the best thresholds derived were 111 cm^3^, 660 and 38 cm respectively (Table 3, Supplemental Fig. 1). In univariate analysis, gender male, advance age (> 65 years), advanced stage (III and IV), IPI score > 2, iPET metabolic response, eotPET metabolic response and MTV, TLG, Dmax were significantly associated with the risk of progression/relapse (Fig. 2; Table 4) while the other features included (bulky disease, B symptoms, LDH value) were not associated. At multivariate analysis, only PET-related variables were confirmed to be independent prognostic factors. Particularly, a nCMR at iPET and eotPET were significantly correlated with short PFS (p value 0.001 and < 0.001, respectively). Also high value of MTV (> 111 cm3), of TLG (> 660) and Dmax (> 38 cm) were confirmed to be significantly correlated (p value = 0.002, 0.009 and 0.011). To develop a more robust and accurate prognostic score, we combined MTV and Dmax to derive three distinct risk categories (TLG was excluded due to its collinearity with MTV, although the latter showed a more significant p-value). These categories were: (1) low MTV (≤ 111 cm³) and low Dmax (≤ 38 cm); (2) low MTV (≤ 111 cm³) + high Dmax (> 38 cm) or high MTV (> 111 cm³) + low Dmax (≤ 38 cm); and (3) high MTV (> 111 cm³) and high Dmax (> 38 cm). As shown in Fig. 3, this system provided superior PFS stratification in our cohort, compared to the single factors. Specifically, patients with both low MTV and low Dmax had a 5-year PFS rate of 90% (12 events out of 174 cases; 7%), those with one high-risk feature had a PFS of 56% (33/91 events; 36%), and patients with both high MTV and high Dmax had a PFS of 39% (63/96 events; 66%) (Fig. 4).

**Table 3:** Receiver operating characteristic (ROC) curve analysis of metabolic baseline 18F-FDG PET/CT parameters

	Threshold	Sensitivity (95%CI)	Specificity (95%CI)	*p* value	AUC (95%CI)
MTV, cm^3^	111	74% (65.5-82)	78% (72-82)	<0.001	0.784 (0.740-0.824)
TLG	660	79% (70-86)	73% (67-78)	<0.001	0.779 (0.735-0.819)
Dmax, cm	38	72% (63-80)	75% (70-80)	<0.001	0.751 (0.705-0.793)

*MTV *total metabolic tumor volume, *TLG *total lesion glycolysis, *Dmax*, maximum tumor dissemination, *AUC *area under curve

**Table 4:** Univariate and multivariate analyses for PFS

	Univariate analysis		Multivariate analysis	
	*p* value	HR (95% CI)	*p* value	HR (95% CI)
Gender male	0.004	1.688 (1.172-2.429)	0.889	1-037 (0.617-1.742)
Age advanced	0.001	2.320 (1.383-3.890)	0.682	0.951 (0.554-1.664)
Stage advanced	<0.001	2.662 (1.882-3.887)	0.644	1.157 (0.662-2.149)
B symptoms	0.501	1.770 (0.800-2.234		
Bulky disease	0.304	1.247 (0.820-1.898)		
IPS score>2	<0.001	2.944 (1.815-4.774)	0.068	1.769 (0.957-3.270)
LDH elevated	0.118	1.300 (0.980-1.540)		
iPET nCMR	<0.001	2.998 (1.934-4.647)	0.001	2.451 (1.399-4.313)
eotPET nCMR	<0.001	5.205 (2.536-10.669)	<0.001	2.895 (1.977-4.239)
MTV	<0.001	7.251 (4.886-10.762)	0.002	3.222 (1.726-6.014)
TLG	<0.001	4.725 (3.269-6.833)	0.009	2.890 (1.200-5.201)
Dmax	<0.001	5.123 (3.479-7.543)	0.011	2.108 (1.152-3.988)

*PFS* progression free survival, *HR* hazard ratio, CI confidence interval; nCMR: not complete metabolic response; iPET: interim PET; eotPET: end-of-treatment PET

MTV and TLG were evaluated individually in the multivariate analysis due to the collinearity relationship

## Discussion

The research into early and accurate non-invasive biomarkers to predict treatment outcome in HL are desirable, especially in an era of personalized-medicine. The findings of this study underscore the significant prognostic value of baseline 2-[^18^F]FDG PET/CT semiquantitative parameters in patients with HL. By analyzing a large cohort of 361 patients, we demonstrated that metrics reflecting both tumor burden (MTV and TLG) and disease dissemination (Dmax) are independent predictors of chemotherapy response and PFS. MTV and TLG were previously investigated as prognostic factors in HL and other lymphoma histotypes with very positive results [5, 12–14]. These parameters reflect at the same time tumor morphological and functional activity of the disease and may be an indirect expression of tumor load. While visual assessment via the Deauville scale remains the standard for response evaluation [2, 3] and also our study confirms this finding, our data suggests that baseline metabolic volume provides critical “pre-treatment” insight that visual scores alone may miss. The detection of pre-treatment predictive/prognostic features may have the big advantage to find early patients not responsive to therapy and/or with bad prognosis, potentially helping in the choice of the most appropriate regimen. Interestingly, while bulky disease (defined by morphological diameter) is traditionally used for risk stratification, it was not significantly associated with PFS in our univariate analysis. This suggests that MTV, which accounts for the total metabolic activity of all lesions, may be a more accurate representation of true tumor burden than a single linear measurement of the largest mass. Bulky is a simple geometrical definition that doesn’t take into account the heterogeneity of the mass and its specific features. Even the potential usefulness of radiotherapy in bulky disease seems to be uncertain [15]. However, tumor burden features did not consider in their definition the pattern of distribution of disease and/or the number of lesions. Disease dissemination, captured by Dmax, emerged as a powerful prognosticator in our study. While studies on Dmax in HL have previously yielded controversial or limited results, our findings align with emerging evidence suggesting that the spatial distribution of lesions is as critical as their volume [16–18]. High Dmax values (> 38 cm) were significantly correlated with shorter PFS. This indicates that patients with widespread disease across different anatomical compartments face a higher risk of treatment failure, potentially reflecting a more aggressive biological phenotype. Therefore, a metric that quantifies the maximum distance between lesions, such as Dmax, could offer a unique and intuitive reflection of disease spread, potentially correlating with more aggressive tumor biology and poorer outcomes [19]. Dmax prognostic role was previously demonstrated especially in DLBCL [19, 20], while in HL the evidence is still preliminary. Durmo et al. [8] demonstrated that Dmax is independently associated with PFS for early or advanced HL patients treated with ABVD, and similar results derived also by the research of Kanoun et al. [21] in advanced HL patients treated with escalated BEACOPP.

One of the most compelling aspects of this research is the superior risk stratification achieved by combining MTV and Dmax. We observed an outstanding difference in outcomes between the high -risk group (high MTV and Dmax) and the low-risk group (low MTV and low Dmax) achieved a median PFS of 39% vs. 90% (*p* < 0.001). The integration of MTV and Dmax into a simple, clinically intuitive model (low/low, discordant, high/high) successfully identifies a subgroup with an excellent prognosis (5-year PFS 90%) who might be candidates for de-escalation strategies, and a very high-risk subgroup (5-year PFS 39%) for whom treatment intensification or novel therapeutic approaches should be considered. This combined approach addresses two distinct dimensions of the disease: the total amount of metabolic tumor tissue and its spread throughout the body. This dual-metric model could potentially identify “ultra-high-risk” patients at the time of diagnosis who might benefit from more intensive frontline therapies or closer monitoring. Also in another study [9] based on a small population of HL (*n* = 52), the combination of MTV and Dmax expressed as tMTV/DmaxVox, was the only prognostic variable in predicting relapse or progression. Moreover, the combination of MTV and Dmax demonstrated to be a good prognosticator also in other tumors, like prostate cancer [22].

Our findings build upon and refine previous research, such as the work by Mouheb et al. [6], who identified only IPS and TLG as independent prognostic factors in a population of 166 HL patients receiving escalated BEACOPP. However, their study was affected by a relatively smaller sample size, the inclusion of stage I HL as well and fewer recorded events (*n* = 27) compared to the high number of covariates included, which may have impacted the statistical power of their multivariate models. By utilizing a larger cohort and longer follow-up, our study provides more robust evidence for considering the integration of these biomarkers into clinical practice. Though these varied parameters offer encouraging prognostic insights for PFS and treatment response evaluation, their clinical impact is currently limited due to the lack of universal standardization of their measurement and the establishment of shared methodology [23]. In this analysis, we decided to extract MTV and TLG applying a segmentation method with a SUV threshold ≥ 4.0 as suggested by an international consensus [23].

Despite the promising results, this study has limitations. Its retrospective, single-center nature may introduce selection bias, like the fact that patients with positive interim PET switched from ABVD to BEACOPP, which is a potential counfonder but reflects real world clinical practice. Firstly, the retrospective design over a long period (2007–2023) means that PET/CT technology reconstruction protocols, and even first-line treatment standards (e.g., introduction of BV-AVD) evolved, which could introduce heterogeneity. While we used a consistent segmentation method (SUV ≥ 4), manual adjustments or interobserver variability in lesion delineation, particularly in regions of physiological uptake, were not assessed. Secondly, the exclusion of stage I patients limits the generalizability of our Dmax findings to all HL cohorts. Finally, the proposed combined MTV/Dmax model, while powerful in our cohort, requires external validation in prospective, multi-center settings before clinical implementation, such as the threshold got by ROC curve analyses were derived internally and needs validation in external populations. Patients received mainly ABVD, which represented the standard treatment since the recent introduction of new drug combinations [24–26]. Additionally, while we used specialized software (LIFEx) for semiquantitative analysis, the lack of complete standardization in MTV calculation methods across different centers remains a challenge for widespread clinical implementation. Future prospective, multicenter trials are necessary to validate these thresholds and determine if treatment de-escalation or escalation based on baseline MTV and Dmax can improve long-term patient outcomes, balancing efficacy, safety and cost-effectiveness.

## Conclusion

In conclusion, baseline MTV and Dmax are strong, independent prognostic biomarkers in cHL. Their combination provides superior risk stratification, identifying patients with divergent outcomes. These readily available PET/CT parameters warrant prospective validation to assess their utility in guiding risk-adapted treatment strategies.

## Supplementary Information

Below is the link to the electronic supplementary material.

Supplementary Material 1

## Data Availability

The datasets generated and/or analyzed in the current study are not publicly available due to the approval of the Ethics Committee but are available from the corresponding author on reasonable request.
