# Supplementary figures and images for: The predictive and prognostic role of baseline 2-[18F]FDG PET/CT volumetric and dissemination features in classical Hodgkin lymphoma
Source: Eur J Nucl Med Mol Imaging. 2026 Mar 25;53(9):5243–50. doi: 10.1007/s00259-026-07862-x (PMC13315121; doi:10.1007/s00259-026-07862-x)

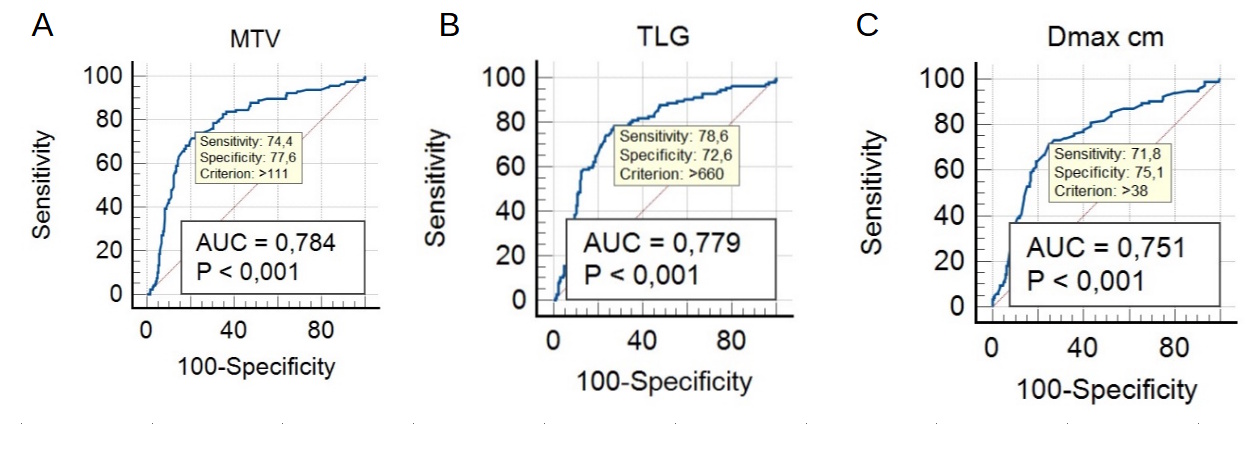

Supplement: Supplementary file 1 — Supplementary Material 1 [file 259_2026_7862_MOESM1_ESM.jpg]
